# Avaliação da hesitação vacinal para a vacina contra a influenza sazonal entre professores da rede pública de Teresina, Piauí, Brasil, em tempos de COVID-19

**DOI:** 10.1590/0102-311XPT167823

**Published:** 2024-11-22

**Authors:** Bruna Luisa Figueirêdo Pierote, Martha Cecília Suárez-Mutis, Guilherme Loureiro Werneck

**Affiliations:** 1 Fiocruz Piauí, Teresina, Brasil.; 2 Instituto Oswaldo Cruz, Fundação Oswaldo Cruz, Rio de Janeiro, Brasil.; 3 Instituto de Medicina Social, Universidade do Estado do Rio de Janeiro, Rio de Janeiro, Brasil.

**Keywords:** Hesitação Vacinal, Vacinas, Vacinas contra Influenza, Professores Escolares, Vaccination Hesitancy, Vaccines, Influenza Vaccines, School Teachers, Vacilación a la Vacunación, Vacunas, Vacunas contra la Influenza, Maestros

## Abstract

Os objetivos deste estudo foram avaliar a frequência de hesitação à vacina contra influenza sazonal e analisar os fatores associados entre professores da rede municipal de ensino de Teresina, Piauí, Brasil. Foi aplicado um questionário *online*, adaptado do Modelo de Crenças em Saúde e foram incluídos 449 docentes. Do público entrevistado, a maioria reside na capital Teresina (373, 83,26%), são mulheres (360, 80,54%), com idade entre 23 e 50 anos (306, 81,38%), naturais do Estado do Piauí (382, 86,82%), de cor parda (289, 64,65%), casados (254, 56,7%) e com pós-graduação *lato sensu* (327, 72,99%). Um total de 33,18% foram considerados hesitantes à vacinação, sendo definidos como hesitantes aqueles professores que não se vacinaram contra influenza em 2020. As variáveis associadas à hesitação nesse grupo foram: não terem se vacinado contra a gripe (influenza) em 2019, acreditarem que a vacina contra a gripe não é conveniente, existem muitos riscos associados à vacina da gripe, e preocupação com reações à vacina da gripe, a pandemia do novo coronavírus mudou a relação com o ato de vacinar e uma maior adesão à vacina após ouvir informações sobre seus benefícios nos meios de comunicação. No entanto, na análise de regressão, somente as variáveis de não se vacinar contra influenza em 2019 e a modificação do comportamento após a pandemia foram significativas. As vacinas seguem sendo o principal método de prevenção e controle de uma série de doenças relacionadas aos vírus da influenza, sendo necessária uma maior adesão por parte dos professores, público prioritário que está em constante contato com alunos de diversas origens e representam uma importante fonte de disseminação do vírus.

## Abstract (en)

The objectives of this study were to evaluate the frequency of vaccine hesitancy against seasonal influenza and to analyze the associated factors among teachers in the municipal school system of Teresina, Piauí State, Brazil. An online questionnaire was applied, adapted from the Health Belief Model, and 449 teachers were included. Of the public interviewed, most live in the capital Teresina, (373, 83.26%), are women (360, 80.54%), aged between 23 and 50 years (306, 81.38%), born in the state of Piauí (382, 86.82%), brown (289, 64.65%), married (254, 56.70%) and with a lato sensu graduate degree (327, 72.99%). In total, 33.18% were considered hesitant, and hesitancy was defined as teachers who were not vaccinated against influenza in 2020. The variables associated with hesitancy in this group were: not being vaccinated against influenza in 2019, the flu vaccine is not convenient, there are many risks associated with it, and concern about reactions to the flu vaccine, the new coronavirus pandemic changed the relationship with the act of vaccinating and greater adherence to the vaccine after hearing information about its benefits in the media. However, in the regression analysis, only the variables “not being vaccinated against influenza in 2019” and “the change in behavior after the pandemic” were significant. Vaccines continue to be the main method of prevention and control of a series of diseases related to influenza viruses, requiring greater adherence by teachers, a priority population that is in constant contact with students from different backgrounds, representing an important source of virus dissemination.

## Resumen

Los objetivos de este estudio fueron evaluar la reticencia a la vacunación contra la influenza estacional y analizar los factores asociados entre los profesores de las escuelas municipales de Teresina, Piauí, Brasil. Se aplicó un cuestionario en línea adaptado del Modelo de Creencias en Salud y se incluyeron a 449 profesores. La mayoría de los participantes viven en la capital Teresina (373, 83,26%), son mujeres (360, 80,54%), de entre 23 y 50 años de edad (306, 81,38%), nacidos en el estado de Piauí (382, 86,82%), de color pardo (289, 64,65%), casados (254, 56,70%) y con título de posgrado (327, 72,99%). El 33,18% del total se consideró reticente a la vacunación, y se definió como reticentes a aquellos profesores que no se vacunaron contra la gripe en 2020. Las variables asociadas a la reticencia fueron: no haberse vacunado contra la gripe (influenza) en 2019; no considerar oportuna la vacuna contra la gripe; considerar muchos riesgos asociados a la vacuna contra la gripe y preocuparse por las reacciones a esta vacuna. La pandemia del nuevo coronavirus impactó la relación entre el acto de vacunarse y una mayor adherencia a la vacunación tras enterarse de sus posibles beneficios en los medios de comunicación. En el análisis de regresión, solo las variables no vacunarse contra la influenza en 2019 y el cambio del comportamiento después de la pandemia fueron significativas. La vacunación sigue siendo el principal método de prevención y control de enfermedades relacionadas con los virus de la gripe, lo que requiere una mayor adherencia por parte de los profesores, un público prioritario que está en constante contacto con los estudiantes de distintos orígenes y que representa una importante fuente de propagación del virus.

## Introdução

A hesitação à vacinas é elencado como um dos maiores desafios de Saúde Pública global, uma vez que o atraso nos programas de imunização gera uma sobressaturação desnecessária de pacientes em sistemas de saúde por conta de doenças evitáveis pela vacina, como as síndromes respiratórias causadas pelo vírus da influenza ^1^ ^,^ ^2^ ^,^ ^3^. A diminuição das taxas de cobertura vacinal em todas as faixas etárias teve como consequência a volta de doenças que haviam sido eliminadas em vários territórios, por serem doenças evitáveis por imunizantes, como o sarampo e a rubéola ^4^ ^,^ ^5^, representando um retrocesso em vários aspectos para os sistemas de saúde. Alguns dos fatores que influenciam na diminuição das taxas de cobertura vacinal são notícias sobre possíveis efeitos colaterais das vacinas, experiências anteriores ruins e a hesitação vacinal ^6^. Com a deflagração da pandemia do novo coronavírus, as taxas de cobertura vacinal em todo o mundo foram ainda mais impactadas ^7^ ^,^ ^8^.

Um dos principais motivos da queda nas taxas de cobertura vacinal é, exatamente, o fenômeno global de hesitação à vacina ^9^ ^,^ ^10^ ^,^ ^11^. Hesitar não significa necessariamente não vacinar, mas atrasar ou recusar o ato de imunização, mesmo com o recurso disponível ^12^, ação que tem sido cada vez mais expandida na população, inclusive entre profissionais de saúde, causando muitos empecilhos para as metas de imunização em diferentes sistemas de saúde ^13^.

A vacina contra influenza é distribuída gratuitamente no Brasil para professores de escolas públicas e privadas desde 2018 pelo Sistema Único de Saúde (SUS) ^14^ e a adesão para cumprimento da meta de cobertura de pelo menos 90% dessa população é essencial para monitoramento e ampliação dos programas de prevenção. Vacinar o público de professores é considerado prioridade em alguns países ^15^, pelo fato do ambiente de trabalho, a sala de aula, ser um local de alta transmissão de vírus ^16^ ^,^ ^17^ ^,^ ^18^ ^,^ ^19^. Esse público pertence a uma categoria importante de formadores de opinião, especialmente no ambiente escolar, e estimar a frequência da hesitação vacinal, assim como os possíveis fatores que levam a essa conduta, é relevante para a formulação de políticas públicas que pretendam aumentar a cobertura vacinal em diferentes grupos sociais. Uma vez que há poucos estudos sobre este tema relacionado aos professores, este trabalho teve como objetivo estudar a frequência de hesitação à vacina contra influenza sazonal e analisar os fatores associados e sua interferência na adesão à vacina entre professores da rede municipal de ensino de Teresina, no Piauí.

## Métodos

### Desenho do estudo

Este estudo tem como base uma pesquisa observacional transversal a partir de um questionário online, previamente validado e aplicado a professores da rede pública da Secretaria Municipal de Educação de Teresina (SEMEC).

### População de estudo e amostra

O Município de Teresina tem um território de 1.391.293km^2^, população de aproximadamente 871.126 habitantes e 304 escolas municipais, 173 estaduais, 191 privadas e 4 federais. No município, há cadastrados 3.389 professores nos ensinos infantil e fundamental. O cálculo do tamanho amostral foi baseado numa frequência de 50%, margem de erro de 5%, efeito de desenho de 1 e uma confiabilidade de 95%. O tamanho mínimo de amostra previsto foi de 347 participantes.

### O instrumento de pesquisa

Como instrumento de pesquisa, foi utilizado o formulário adaptado de Neves ^20^ para avaliar a adesão à vacinação com base no Modelo de Crenças em Saúde (MCS) utilizado por Shahrabani & Benzion ^21^ e Blue & Valley ^22^. Esse instrumento avalia sete dimensões: Suscetibilidade à influenza, Gravidade da influenza, Benefícios da vacina, Barreiras de aceitação à vacina, Estímulos para ação, Conhecimento e Motivação para a saúde (Quadro 1). Devido à pandemia do novo coronavírus surgir antes da etapa de aplicação do questionário, foram acrescentadas algumas questões associadas ao novo vírus e outras para coleta do perfil sociodemográfico dos entrevistados. Ao final, o questionário tinha setenta variáveis. Portanto, devido à nova adaptação do questionário, foi realizado um estudo piloto para validar o instrumento com um público menor, de apenas trinta professores da Universidade Estadual do Piauí. Essas respostas foram consolidadas, o questionário foi adequado e as informações obtidas não foram usadas neste estudo.

**Quadro 1:** Características de hesitação vacinal baseada no Modelo de Crenças em Saúde (MCS).

DIMENSÃO	VARIÁVEIS
Suscetibilidade à influenza	1. Trabalhar com muitas pessoas todo dia aumenta minhas chances de pegar gripe 2. Apenas pessoas com mais de 60 anos de idade pegam gripe 3. Eu tenho grande chance de pegar gripe 4. Pessoas saudáveis podem pegar gripe 5. Eu acho que minha chance de pegar gripe no futuro é grande 6. Eu me preocupo muito com a possibilidade de pegar gripe 7. Eu vou pegar gripe no próximo ano
Gravidade da influenza	1. Pensar que posso pegar gripe me assusta 2. Se eu pegasse gripe, poderia perder meu emprego 3. Se eu pegasse gripe, isso prejudicaria a minha família 4. Estar gripado tornaria as atividades diárias mais difíceis 5. Se eu pegasse gripe, isso seria mais grave do que outras doenças 6. A gripe pode ser uma doença grave
Benefícios da vacina	1. Vacinar-me contra gripe vai me impedir de pegar gripe 2. Vacinar-me contra gripe vai proteger as pessoas que moram comigo de pegarem gripe 3. Vacinar-me contra gripe vai me impedir de faltar ao trabalho 4. Eu tenho muito a ganhar ao me vacinar contra gripe 5. Eu não teria medo de pegar gripe se eu me vacinasse contra gripe 6. Ter uma doença crônica (como diabetes, doença do coração ou asma) é um motivo para se vacinar contra gripe
Barreiras de aceitação à vacina	1. Vacinar-me contra gripe não é conveniente para mim 2. Para me vacinar contra gripe, eu precisaria abrir mão de muitas coisas 3. Vacinar-me contra gripe pode ser doloroso 4. Vacinar-me contra gripe é demorado 5. Vacinar-me contra gripe interfere nas minhas atividades diárias 6. Existem muitos riscos associados à vacina da gripe 7. A vacina da gripe custa caro demais 8. Fico preocupado em ter uma reação à vacina da gripe
Estímulos para a ação	1. Eu decidi me vacinar contra gripe quando vi um anúncio da campanha 2. Eu me vacinei contra gripe porque um amigo ou familiar me disse que era importante 3. Eu me vacinei contra gripe porque um profissional de saúde me recomendou 4. Eu me vacinei contra gripe porque meu chefe achou que seria uma boa ideia 5. Eu me vacinei contra gripe após ouvir informações sobre os benefícios da vacina nos meios de comunicação
Conhecimento	1. As pessoas ficam gripadas por comer/beber algo de outras pessoas gripadas 2. As pessoas pegam gripe ao respirar o mesmo ar que outras pessoas gripadas 3. A gripe dura de três a cinco dias 4. A gripe pode causar uma doença mais grave, como pneumonia 5. Uma pessoa pode pegar gripe ao se vacinar contra gripe 6. Pessoas muitas vezes ficam doentes ao se vacinarem contra gripe
Motivação para a saúde	1. Eu tenho uma alimentação balanceada 2. Eu sigo as orientações médicas porque acredito que elas beneficiarão o meu estado de saúde 3. Eu frequentemente faço coisas por vontade própria para melhorar a minha saúde 4. Eu pesquiso novas informações relacionadas com a minha saúde 5. Eu faço exames preventivos periódicos além de ir ao médico quando necessário 6. Eu faço exames odontológicos periódicos além de visitas para problemas específicos 7. Eu faço exercícios físicos regularmente, ao menos 3 vezes por semana

Fonte: adaptado de Neves ^20^.

### Critérios de inclusão e exclusão

Foram incluídos no estudo professores de qualquer sexo, de faixa etária acima de 18 anos, cadastrados na SEMEC, de todos os níveis da educação básica (Educação Infantil, Ensino Fundamental e Ensino Médio), que após serem informados do estudo, concordaram em assinar o Termo de Consentimento Livre e Esclarecido (TCLE) e enviaram o formulário preenchido via plataforma Google Forms (https://docs.google.com/forms). Foram excluídos os participantes que não preencheram adequadamente o questionário, seja por ter respostas em duplicata ou não respondentes. Apenas um participante, de acordo com a data de nascimento preenchida no formulário, tinha idade abaixo de 18 anos e foi excluído da análise dos dados. Outros oito participantes foram excluídos pelos motivos assinalados anteriormente.

### Inclusão dos participantes

Foi enviado um e-mail com o convite para participar da pesquisa a todos os 3.389 professores cadastrados na SEMEC, com o TCLE e o formulário eletrônico. Com apoio da SEMEC, os e-mails foram enviados de forma individual a cada participante e foi possível contar com 474 respostas no sistema de formulário enviado (plataforma Google Forms), das quais, com a retirada de respostas em duplicata e não respondentes à variável desfecho, resultaram 449 professores incluídos.

### Análise dos dados

Foram criadas tabelas de frequências para todas as respostas do formulário. As tabelas de frequências são uma forma de análise unidimensional de uma variável com categorias ^23^ determinadas. As análises realizadas com os dados obtidos do questionário tiveram como variável de interesse a questão “Você vacinou contra influenza em 2020?”. Não ter vacinado contra influenza em 2020 foi considerado como “hesitação à vacinação contra influenza”. O início do ano de 2020 foi o momento em que foi deflagrada a pandemia do novo coronavírus e os professores de escolas públicas e particulares já faziam parte do grupo prioritário para receber a vacina contra influenza, gratuitamente, durante a campanha nacional ofertada pelo SUS. A análise unidimensional realizada está relacionada às 149 respostas à pergunta “Você vacinou contra influenza em 2020?” ditas “Não”. Todas as análises foram realizadas no ambiente R (http://www.r-project.org), utilizando as bibliotecas *dplyr*, *psych* e *readxl*. Nas análises bivariadas, as variáveis foram dispostas na forma de tabelas e um teste qui-quadrado de independência foi feito para avaliar se cada pergunta do formulário tem alguma associação com a variável de interesse. Uma outra base de dados foi criada com as variáveis que foram selecionadas para serem usadas em modelos de regressão logística, sendo que a distribuição escolhida para a variável resposta foi a distribuição binomial. Dois modelos foram propostos, e a distribuição escolhida para a variável resposta foi a distribuição binomial. No modelo 1, foram consideradas as variáveis que se mostraram associadas à variável de interesse “Você vacinou contra influenza em 2020?” com um nível de significância de até 0,1. No modelo 2 foram consideradas as variáveis que se mostraram associadas à variável de interesse “Você vacinou contra influenza em 2020?” com um nível de significância de até 0,1 ou próximo (valores entre 0,1 e 0,15). O modelo proposto foi:

$$y=ln\left(Ρ∕\left(1-Ρ\right)\right)=b0+b1Χ1+\ldots +biΧi$$

sendo, P: probabilidade de um desfecho ocorrer; 1 - P: probabilidade de um desfecho não ocorrer; (P/(1 - P)): transformação *logit* variável dependente; b0: intercepto; bi: preditores; Xi: variáveis explicativas.

### Aspectos éticos

Esta pesquisa foi submetida à avaliação pelo Comitê de Ética em Pesquisa com Seres Humanos do Instituto Oswaldo Cruz, Fundação Oswaldo Cruz (IOC/FIOCRUZ; CAAE: 40710020.1.0000.5248, parecer nº 4.549.414 de 21/02/2021).

## Resultados

Em total, foram incluídos 449 professores, dos quais 149 (33,18%) não se vacinaram contra influenza em 2020. Entre os professores incluídos 373 (83,26%) são residentes da capital Teresina, incluindo as áreas urbana e rural; 75 (16,14%) residem em outras cidades. A maioria dos professores respondentes foram mulheres (360, 80,54%), com idade entre 23 e 67 anos (306, 81,38%; média: 41,28 ± 8,66; mediana: 40 anos, intervalo interquartil - IIQ: 35-47), naturais do Estado do Piauí (382, 86,82%), de cor parda (289, 64,65%) e casados (254, 56,7%). Do total, 327 (72,99%) tem como escolaridade máxima um curso de especialização ou residência (pós-graduação *lato sensu*), 26 (5,8%) tinham feito mestrado ou doutorado. Entre os professores que hesitaram em se vacinar em 2020, 97 (65,1%) haviam concluído um curso de especialização e 52 (34,9%) haviam concluído somente o curso superior ou haviam feito um curso *stricto sensu* (*odds ratio* - OR = 0,56; intervalo de 95% de confiança - IC95%: 0,36-0,86; p = 0,008). Os dados sociodemográficos sobre os professores participantes da pesquisa estão descritos na Tabela 1.

**Tabela 1:** Características sociodemográficas dos professores de escola pública municipal de Teresina, Piauí, Brasil, 2021, com relação à hesitação a vacinação contra influenza em 2020.

Características	Total	Vacinou contra influenza em 2020		Valor de p *
		Não (%)	Sim (%)	
Total	449	149 (33,18)	300 (66,82)	
Sexo				
Feminino	360	114 (76,51)	246 (82,55)	0,128
Masculino	87	35 (23,49)	52 (17,45)	
Faixa etária (anos)				
23-50	306	98 (32,03)	208 (67,97)	0,084
> 50	70	30 (42,86)	40 (57,14)	
Naturalidade				
Piauí	382	124 (85,52)	258 (87,46)	0,572
Outro	58	21 (14,48)	37 (12,54)	
Raça/Cor				
Pardo	289	99 (66,44)	190 (63,76)	0,789
Preto	71	25 (16,78)	46 (15,44)	
Branco	66	19 (12,75)	47 (15,77)	
Outro	21	6 (4,03)	15 (5,03)	
Estado civil				
Casado(a)	254	77 (51,68)	177 (50,20)	0,150
Solteiro(a)	148	53 (35,57)	95 (31,77)	
Separado(a) ou divorciado (a)	39	18 (12,08)	21 (7,02)	
Viúvo(a)	7	1 (0,67)	6 (2,01)	
Escolaridade				
Ensino Superior	95	41 (27,52)	54 (18,06)	0,029
Pós-graduação - *lato sensu*	327	97 (65,10)	230 (76,92)	
Pós-graduação - *stricto sensu*	26	11 (7,38)	15 (5,02)	
Tem filho(s)				
Sim	298	94 (63,0)	204 (68,23)	0,277
Não	150	55 (36,91)	95 (31,77)	
Residência atual				
Teresina	373	131 (87,92)	242 (80,94)	0,062
Outra cidade	75	18 (12,08)	57 (19,06)	

* Teste qui-quadrado de independência.

A Tabela 2 mostra as informações sobre o ato de vacinar contra influenza e COVID-19. Em total, 266 professores (61,14%) se vacinaram contra influenza tanto em 2019 como em 2020; 58 (13,33%) dos professores não se vacinaram em 2019 nem em 2020. A chance de hesitar em 2020 entre os professores que não se vacinaram em 2019 foi 7,39 vezes maior que aqueles vacinados nesse ano (OR = 7,39; IC95%: 4,33-12,60; p ≤ 0,01). Entre aqueles que hesitaram, 17 (11,64%) concordam apenas parcialmente que a vacina é um importante método de controle e prevenção de doenças. A chance de hesitar entre aqueles professores que concordam parcialmente na importância da vacinação foi 2,33 vezes maior (IC95%: 1,14-4,76; p = 0,017) comparado aos que não hesitaram em se vacinar em 2020. Outro resultado é que entre os que hesitaram, 66 (44,9%) acreditam que a pandemia do novo coronavírus mudou a relação com o ato de vacinar, comparado com 62 (20,74%) dos que não hesitaram (p < 0,001). A chance de mudar de opinião sobre a importância da vacinação após a pandemia entre os que hesitaram foi 3,11 vezes maior (IC95%: 2,03-4,78) comparada aos que não hesitaram.

**Tabela 2:** Informações sobre o ato de vacinar contra influenza e COVID-19 de professores do ensino municipal de Teresina, Piauí, Brasil, com relação à hesitação da vacinação da influenza em 2020.

Características	Total	Hesitou em vacinar em 2020		OR (IC95%)	Valor de p *
		Sim (%)	Não (%)		
Total	449	149 (33,18)	300 (66,82)		
Vacinou contra gripe (influenza) em 2019					
Não	82	58 (40,00)	24 (8,28)	7,39 (4,33-12,60)	< 0,001
Sim	353	87 (60,00)	266 (91,72)		
Concorda que a vacina é um importante método de controle e prevenção de doenças					
Concorda parcialmente	33	17 (11,64)	16 (5,35)	2,33 (1,14-2,36)	0,017
Concorda totalmente	412	129 (88,36)	283 (94,65)		
A pandemia do novo coronavírus mudou a relação com o ato de vacinar					
Sim	128	66 (44,90)	62 (20,74)	3,11 (2,03-4,78)	< 0,001
Não	318	81 (55,10)	237 (79,26)		
Vacinou contra o novo coronavírus					
Não	446	147 (99,32)	299 (99,67)	0,49 (0,03-7,92)	0,61
Sim	2	1 (0,68)	1 (0,33)		
Convive com pessoas que fazem parte do grupo de risco					
Sim	379	121 (81,76)	258 (86,00)	0,72 (0,43-1,24)	0,242
Não	69	27 (18,243)	42 (14,00)		

Nota: muitas somas não conferem porque não havia respostas para todos os itens citados.

* Teste qui-quadrado de independência.

Em relação às questões de dimensões de hesitação à vacinação (Suscetibilidade à influenza, Gravidade da influenza, Benefícios da vacina, Barreiras de aceitação à vacina, Estímulos para ação, Conhecimento e Motivação para a saúde) adaptadas do questionário de Neves ^20^, foram apontadas algumas associações interessantes, como apresentado na Tabela 3. Na dimensão Benefícios da vacina, 8,05% dos que hesitaram e 2,01% dos que não hesitaram discordam que tem muito a ganhar ao se vacinar contra a gripe (p < 0,01).

**Tabela 3:** Características de hesitação a vacinas segundo as dimensões do Modelo de Crenças na Saúde.

Características	Total	Hesitou em vacinar em 2020		OR (IC95%)	Valor de p *
		Sim (%)	Não (%)		
Total	449	149 (33,18)	300 (66,82)		
Dimensão Beneficios da vacina					
Eu tenho muito a ganhar ao me vacinar contra a gripe					
Discordo	18	12 (8,05)	6 (2,01)	4,28 (1,57-11,64)	0,002
Concordo	430	137 (91,95)	293 (97,99)		
Dimensão Barreiras de aceitação à vacina					
Vacinar-me contra gripe não é conveniente para mim					
Concordo	29	20 (13,70)	9 (3,01)	5,11 (2,26-11,54)	0,00002
Discordo	416	126 (86,30)	290 (96,99)		
Vacinar-me contra gripe é demorado					
Concordo	96	42 (28,19)	54 (18,06)	1,78 (1,12-2,83)	0,014
Discordo	352	107 (71,81)	245 (81,94)		
Vacinar-me contra gripe interfere nas minhas atividades diárias					
Concordo	39	20 (13,51)	19 (6,35)	2,30 (1,19-4,46)	0,012
Discordo	408	128 (86,49)	280 (93,64)		
Existem muitos riscos associados à vacina da gripe					
Concordo	74	35 (23,49)	39 (13,04)	2,04 (1,23-3,40)	0,005
Discordo	374	114 (76,51)	260 (86,96)		
Fico preocupado em ter uma reação à vacina da gripe					
Concordo	154	69 (46,94)	85 (28,33)	2,24 (1,49-3,37)	0,0001
Discordo	293	78 (53,06)	215 (71,67)		
Dimensão Estímulos para ação					
Eu me vacinei contra gripe após ouvir informações sobre os benefícios da vacina nos meios de comunicação					
Discordo	74	37 (25,34)	37 (12,50)	2,38 (1,43-3,95)	< 0,0006
Concordo	368	109 (74,66)	259 (87,50)		
Dimensão Motivação para a saúde					
Eu sigo as orientações médicas porque acredito que elas beneficiarão o meu estado de saúde.					
Discordo	38	20 (13,42)	18 (6,06)	2,40 (1,23-4,69)	0,009
Concordo	408	129 (86,58)	279 (93,94)		
Eu faço exames preventivos periódicos além de ir ao médico quando necessário					
Discordo	58	28 (18,92)	30 (10,03)	2,09 (1,19-3,66)	0,008
Concordo	389	120 (81,08)	269 (89,97)		

IC95%: intervalo de 95% de confiança; OR: *odds ratio*.

Nota: muitas somas não conferem porque não havia respostas para todos os itens citados.

* Teste qui-quadrado de independência.

Na dimensão Barreiras de aceitação à vacina, entre os que hesitaram, 13,7% concordam em que se vacinar contra a influenza não é conveniente comparado com 3,01% dos vacinados que concordam com afirmação. Essas diferenças foram estatisticamente significativas (p < 0,001). Da mesma forma, entre os professores que hesitaram, 28,19% apontaram que se vacinar contra a gripe é um processo demorado, comparado com 18,06% dos que não hesitaram (p = 0,014). Ao afirmar que a vacinação interfere nas atividades diárias, 13,51% dos hesitantes concordam, em comparação, entre os docentes que não hesitam, 6,35% concordam com essa afirmação. Essas diferenças também foram estatisticamente significativas (p = 0,012). Quanto à asseveração de que existem muitos riscos associados à vacina da gripe, 23,49% do grupo dos que hesitam em se vacinar, concordam, comparado com 13,04% do grupo dos que não hesitam em vacinar. Essas diferenças também foram estatisticamente significativas (p = 0,005). Finalmente, entre os que hesitam em se vacinar, 46,94% concordam que ficam preocupados em ter uma reação adversa à vacina, comparado com 28,33% dos que não hesitam; essas diferenças também foram estatisticamente significativas (p = 0,001) colaborando com os achados de Moore et al. ^24^, que em estudo realizado com pessoas de todo o Brasil, encontrou que os hesitantes tinham medo de reação adversa à vacina.

Na dimensão Estímulos para ação, aqueles que acreditam que ouvir informações sobre os benefícios da vacina nos meios de comunicação influenciou na chance de se vacinar, 74,66% dos hesitantes concordaram com essa afirmação, comparado com 87,5% dos não hesitantes. Essas diferenças foram estatisticamente significativas (p < 0,01).

Na dimensão Motivação para a saúde, 13,42% dos hesitantes e 6,06% dos que não hesitaram discordam que seguir orientações médicas beneficiam seu estado de saúde; essas diferenças foram estatisticamente significativas (p < 0,01). Ainda nessa dimensão, 18,92% dos hesitantes e 10,03% dos que não hesitaram, não fazem exames preventivos periódicos além de não ir ao médico quando necessário; essas diferenças também foram estatisticamente significativas (p < 0,01). Ao realizar o modelo binomial da regressão logística, somente duas variáveis se mostraram significativas: não ter se vacinado contra influenza em 2019 (OR = 4,89, IC95%: 2,51-9,81) e acreditar que a pandemia pelo novo coronavírus tenha mudado a sua percepção sobre o ato de se vacinar (OR = 3,84; IC95%: 2,14-3,97) (Tabela 4).

**Tabela 4 Modelo:** binomial das variáveis respostas selecionadas e a associação delas com a hesitação a vacinação contra influenza em 2020.

Variável	OR	5%	95%
Faixa etária (anos)			
23-50	0,719	0,351	1,503
Escolaridade			
Ensino Superior	1,528	0,77	3,008
Pós-graduação - *stricto sensu*	1,144	0,305	3,856
Residência atual			
Outro	0,61	0,26	1,347
Vacinou contra gripe (influenza) em 2019			
Não	4,897	2,512	9,811
Concorda que a vacina é um importante método de controle e prevenção de doenças			
Concordo parcialmente	2,635	0,871	8,251
A pandemia do novo coronavírus mudou a relação com o ato de vacinar			
Sim	3,84	2,14	3,97
Convivo com diabético			
Não	0,496	0,274	0,892
A gripe pode ser uma doença grave			
Concordo	0,657	0,313	1,393
Eu tenho muito a ganhar ao me vacinar contra a gripe			
Concordo	0,324	0,054	1,581
Vacinar-me contra gripe não é conveniente para mim			
Concordo	1,559	0,475	4,893
Vacinar-me contra gripe é demorado			
Concordo	1,225	0,622	2,367
Vacinar-me contra gripe interfere nas minhas atividades diárias			
Concordo	1,61	0,599	4,351
Existem muitos riscos associados à vacina da gripe			
Concordo	0,956	0,374	2,373
Fico preocupado em ter uma reação à vacina da gripe			
Concordo	1,137	0,607	2,098
Eu decidi me vacinar contra gripe quando vi um anúncio da campanha			
Concordo	0,736	0,405	1,343
Eu me vacinei contra gripe após ouvir informações sobre os benefícios da vacina nos meios de comunicação			
Concordo	1,18	0,519	2,821
A gripe pode causar uma doença mais grave, como pneumonia			
Concordo	0,521	0,063	3,901
Eu tenho uma alimentação balanceada			
Concordo	0,761	0,409	1,422
Eu sigo as orientações médicas porque acredito que elas beneficiarão o meu estado de saúde			
Concordo	0,945	0,364	2,499
Eu faço exames preventivos periódicos além de ir ao médico quando necessário			
Concordo	0,864	0,349	2,226
Sexo			
Feminino	1,609	0,75	3,619
Estado civil			
Casado	0,482	0,175	1,362
Solteiro(a)	0,65	0,23	1,878
Viúvo(a)	0,0	-	-
Convivo com gestante			
Sim	1,311	0,391	4,796
Pensar que posso pegar gripe me assusta			
Concordo	0,864	0,431	1,771
Uma pessoa pode pegar gripe ao se vacinar contra gripe			
Concordo	1,255	0,701	2,293
AIC	312,23		

AIC: critério de informação de Akaike; OR: *odds ratio*.

## Discussão

No Brasil, a vacinação contra o vírus influenza foi incorporada no Programa Nacional de Imunizações (PNI) no ano de 1999, com o objetivo de diminuir a carga da doença e prevenir as complicações associadas nos grupos prioritários reduzindo assim, a sobrecarga nos serviços de saúde ^25^ ^,^ ^26^. No Brasil, crianças entre 6 meses e menores de 6 anos de idade, gestantes, puérperas, populações indígenas, trabalhadores do setor da saúde, professores das escolas públicas e privadas, indivíduos com doenças crônicas, pessoas com deficiência permanente, entre outros, conformam o grupo alvo da vacinação. A hesitação à vacinação tem sido elencada como uma das 10 principais ameaças à saúde pública global pela Organização Mundial da Saúde (OMS) ^3^ e entender suas causas nos diferentes grupos é fundamental para estabelecer estratégias que aumentem a adesão da população às vacinas. Recentemente no Brasil, foi adaptado um instrumento de avaliação da hesitação vacinal contra o vírus da influenza ^20^. Nós utilizamos um questionário adaptado de Neves ^20^, acrescido de algumas questões adicionais sobre o perfil sociodemográfico, informações sobre o trabalho e a relação com o ato de vacinar contra a influenza para avaliar a hesitação entre professores da rede pública municipal de ensino em uma capital do Nordeste brasileiro, por ser um público recentemente incorporado na estratégia nacional de vacinação contra o vírus influenza e porque vacinar esse grupo é fundamental não somente para sua própria proteção, mas também das crianças e adolescentes com as quais têm contato na sala de aula, assim como pelo fato de os professores serem formadores de opinião dentro do ambiente escolar ^27^ e na realização de atividades educativas. Com a deflagração da pandemia de COVID-19 no decorrer do trabalho e antes da aplicação do formulário, adicionamos outras perguntas relacionadas com o ato de se vacinar contra o SARS-CoV-2.

Nossos resultados mostraram que, entre 449 professores incluídos no estudo, 149 (33,18%) hesitaram em se vacinar contra a influenza em 2020. De forma complementar, 66,82% professores informaram terem sido vacinados nesse ano. Poucos estudos existem na literatura explorando a hesitação ou a adesão à vacinação contra o vírus da influenza entre professores. Uma pesquisa realizada em escolas rurais do Estado da Georgia (Estados Unidos), entre professores e pessoal do *staff*, avaliou os fatores associados à vacinação contra influenza sazonal, foi encontrado que 62% dos entrevistados informaram terem sido efetivamente vacinados ^27^, percentual similar aos nossos achados.

Entre os profissionais de saúde, a hesitação à vacinação contra a influenza também é preocupante no Brasil, como aponta o resultado de estudos realizados nos últimos dois anos no Estado da Bahia ^13^, em que 25% dos trabalhadores de saúde eram hesitantes, e no Estado de São Paulo, com 30,1% dos estudantes de medicina com o calendário de vacina contra a influenza atrasado ^28^.

Nos resultados deste estudo, duas variáveis se mostraram associadas à hesitação à vacinação contra a influenza em 2020, neste público: não ter sido vacinado em 2019 contra esse vírus e mudança de atitude por causa da pandemia pela COVID-19. Achados da literatura mostram que o comportamento prévio de não se vacinar contra diversas doenças evitáveis por meio da vacinação, como gripe (influenza), sarampo e varicela, repete-se com a vacina contra a COVID-19 ^17^ ^,^ ^29^ ^,^ ^30^. O estudo de Neves et al. ^31^, encontrou que, entre profissionais da saúde, o comportamento prévio em relação a outras vacinas estava associado ao ato de se vacinarem. Estudos com aplicação de questionários sobre vacinas e vacinação realizados com adultos de uma clínica odontológica no Brasil em 2018 ^2^, em enfermeiras em Hong Kong (China) sobre a intenção de vacinar contra COVID-19 ^32^ e na população geral da França sobre a intenção de participar voluntariamente dos estudos de vacina contra COVID-19 enviado por meio das redes sociais ^33^, também encontraram associação entre o comportamento anterior em relação à vacinas e a intenção de vacinarem-se, dados apontam que aqueles que já tinham comportamentos favoráveis à vacinação, tendem a não hesitar e vice-versa ^2^ ^,^ ^20^ ^,^ ^31^ ^,^ ^32^ ^,^ ^33^. Nossos resultados mostram que aqueles professores que não se vacinaram contra influenza em 2019, também hesitaram em se vacinar em 2020, sugerindo que, entre os hesitantes, possa existir um padrão de não vacinação.

Os dados de nosso estudo também apontaram que a pandemia do novo coronavírus mudou a relação de professores hesitantes à vacinação com o ato de vacinarem-se, pois a chance de mudar de ideia diante da vacinação por causa da pandemia pela COVID-19 de um indivíduo que hesitou em se vacinar contra a influenza em 2020 foi 3,11 vezes maior (IC95%: 2,03-4,78; p < 0,01) que em indivíduos que não hesitaram. Esse dado é relevante, uma vez que a diminuição de hesitantes é um dos maiores desafios das campanhas de imunização ^2^ ^,^ ^34^ ^,^ ^35^ ^,^ ^36^. Resultados similares foram encontrados entre estudantes de medicina numa província chinesa, eles acreditam que a disseminação da COVID-19 promoveu sua disposição de receber a vacina contra influenza ^37^. No entanto, esses trabalhos divergem de um estudo realizado com estudantes da Carolina do Sul (Estados Unidos) sobre a percepção de riscos sobre a vacina e a aceitação à vacina contra a COVID-19, esse estudo apontou que entre o público que já não aderia ao calendário vacinal, a atitude não alterou com a chegada da vacina para o novo vírus ^38^ e algumas motivações para a intolerância seriam de ordem religiosa, notícias sobre possíveis efeitos colaterais e ameaça ao livre arbítrio ^39^.

Apesar de outras variáveis somente serem encontradas como associadas à hesitação vacinal nas análises bivariadas, é interessante discutir alguns aspectos que podem ajudar a pensar em estratégias direcionadas a diminuir a hesitação dos professores à vacinação. Um estudo transversal realizado com professores de Nova Jérsei (nos Estados Unidos), encontrou um perfil de hesitação maior entre o público de escolas públicas do que de escolas privadas, além de um perfil hesitante entre professores associado à fonte de coleta de informações sobre as vacinas ^40^. A hesitação vacinal entre professores encontrada em outros países como China ^41^ ^,^ ^42^ e Estados Unidos ^43^ também foi associada à inconsistência de informações sobre a vacina e o vírus, e todos os estudos recentes da área concordam sobre a importância dos professores como divulgadores e educadores da informação correta e seu papel de influência para crianças, adolescentes, pais e profissionais interligados às escolas ^40^ ^,^ ^41^ ^,^ ^42^ ^,^ ^43^ ^,^ ^44^.

Neste estudo a dimensão Barreiras de aceitação à vacina (“Vacinar-me contra gripe não é conveniente para mim”, “Vacinar-me contra gripe é demorado”, “Vacinar-me contra gripe interfere nas minhas atividades diárias”, “Existem muitos riscos associados à vacina da gripe”, “Fico preocupado em ter uma reação à vacina da gripe”) foi considerada relevante nas análises. Embora em Teresina existam campanhas de informação incentivando a vacinação nos grupos prioritários, incluídos os professores dos ensinos Básico e Superior, tanto da rede pública quanto da privada, na prática, a vacinação ocorre nas unidades básicas de saúde (UBS) durante o horário escolar, ou, em alguns casos em outros espaços como centros comerciais e terminais de ônibus, porém sempre no horário em que os professores estão na escola. Um estudo realizado com a população do Malawi também aponta a dificuldade de acesso da população às vacinas como um importante elemento para o fenômeno de hesitação à vacinação ^45^. Dessa forma, a extensão do horário da vacinação ou a proposta de levar as equipes de vacina às escolas seria relevante como uma forma de melhorar a cobertura vacinal nesse público alvo, diminuindo assim uma das barreiras encontradas no trabalho.

Entre a dimensão Benefícios da vacina, outras variáveis como “Eu tenho muito a ganhar ao me vacinar contra a gripe”, ou a dimensão Estímulos para ação (“Eu me vacinei contra gripe após ouvir informações sobre os benefícios da vacina nos meios de comunicação”) e a dimensão Motivação para a saúde (“Eu sigo as orientações médicas porque acredito que elas beneficiarão o meu estado de saúde”, “Eu faço exames preventivos periódicos além de ir ao médico quando necessário”), vão de encontro aos achados na literatura sobre a necessidade da informação correta e acessível chegar na população como importante ferramenta de queda da hesitação à vacinação em todos os lugares e públicos ^40^ ^,^ ^44^ ^,^ ^45^.

Entre as limitações deste estudo, está o fator tempo em que foi aplicado o questionário, ainda no início da pandemia de COVID-19, no qual o cenário mundial ainda era incerto em vários aspectos e havia muita informação incorreta, ganhando força nas mídias sociais, além do fato do estudo ter sido aplicado somente com professores da rede municipal de ensino, que pode não ser representativo do público de professores da rede estadual, privada e de Ensino Superior da mesma cidade, Teresina.

Em todo caso, nossos dados apontam a importância de ampliação de estudos na área e a necessidade de resgate e ampliação da divulgação dos benefícios da vacinação e medidas para combater a queda nas coberturas vacinais, sobretudo por parte de um público tão influente quanto o de professores.

## Conclusão

Vários fatores foram identificados como associados à hesitação à vacinação contra influenza, entretanto, somente dois fatores foram confirmados na regressão logística. O comportamento prévio dos professores em relação ao ato de vacinar medido com a variável “não ter vacinado em 2019”, foi associado à hesitação, concordando com outros achados na literatura. A outra variável associada foi a modificação no comportamento entre professores que hesitavam em se vacinar contra a influenza sazonal devido à pandemia da COVID-19. O fato deste estudo ter encontrado 33% de hesitação à vacinação contra a influenza entre professores responsáveis pela educação básica é preocupante, por ser uma população formadora de opinião que pode influenciar diretamente nas práticas de vacinação das crianças e, por outro lado, é um grupo de risco priorizado pelo PNI, por ter contato direto como muitas pessoas na sala de aula. Os professores deveriam promover educação com compromisso e responsabilidade social com esse tema, para que, desde a infância, a população tenha acesso à informação com respaldo científico e, assim, permitir que o Brasil volte a atingir coberturas vacinais adequadas.
